# Effects of an interprofessional care concept in nursing homes evaluated in the SaarPHIR project: A cluster-randomized controlled trial

**DOI:** 10.1371/journal.pone.0321118

**Published:** 2025-05-15

**Authors:** Lisa Tönnies, Viola Zimmer, Alexandra Piotrowski, Thorsten Lehr, Sonja Laag, Juliane Köberlein-Neu

**Affiliations:** 1 Center for Health Economics and Health Services Research – Schumpeter School of Business and Economics, University of Wuppertal, Wuppertal, Germany; 2 Chair of General Practice II and Patient-Centredness in Primary Care, Institute of General Practice and Primary Care, Faculty of Health, Witten/Herdecke University, Witten, Germany; 3 Saarland University, Saarbrücken, Germany; 4 BARMER, Wuppertal, Germany; King Saud University Medical City, SAUDI ARABIA

## Abstract

**Introduction:**

Deficits in interprofessional collaboration can lead to insufficient medical care for nursing home residents, particularly inappropriate hospitalizations. Transfers are stressful for residents, and hospital stays can lead to infections and functional decline. Increasing the role of general practitioners and improving collaboration between professionals may reduce hospitalizations. In an effort to reduce hospitalizations and improve quality of care for nursing home residents, the SaarPHIR project implemented and evaluated a complex intervention which aimed at improving cooperation between general practitioners and nurses. This paper evaluates the effectiveness of an interprofessional care concept in nursing homes.

**Methods:**

A prospective, cluster-randomized controlled trial was conducted in Saarland, Germany, from May 2019 until July 2020 with a 15-months of follow-up, with two parallel groups and a 1:1 randomization at district level to evaluate the effectiveness of the intervention. The six administrative districts of the German federal state of Saarland were selected as randomization clusters to avoid spillover effects. The primary outcome, hospitalization, was assessed using claims data from six health insurers. Analyses were performed using generalized linear mixed models assuming both a Poisson and, for sensitivity analyses, a negative binomial distribution allowing for clustering at the nursing home level. Considering the randomized cluster level in the primary analysis would be the proper approach. However, after careful consideration, an unconventional approach was adopted to ensure the evaluation of the intervention within the complex healthcare system with a pragmatic design. The randomized cluster level was considered in sensitivity analyses. Secondary outcomes included ambulatory care-sensitive and nursing home care-sensitive admissions, mortality and hospital days. Furthermore, health economic aspects were explored by comparing costs between groups descriptively and exploratively using a generalized linear mixed model with a log-link and a gamma distribution.

**Results:**

Twenty-eight nursing homes received the intervention (1,053 residents), and 16 nursing homes (680 residents) were assigned to usual care. Hospitalization rates did not differ significantly between groups (incidence rate ratio [IRR] = 0.94; 95% CI: 0.78–1.14). Nursing home care-sensitive admissions could be reduced in residents treated with the interprofessional care concept (IRR: 0.73, 95% CI: 0.59–0.96). No differences in mortality, number of days spent in hospital and healthcare costs were found between groups. Mean drug costs (€82.53; 95% CI: 11.79–165.06) were higher and costs for ambulatory hospital stays lower (−€40.80; 95% CI: −76.50–0.00) in the intervention group.

**Conclusion:**

All-cause hospitalization was not significantly affected in the relatively short duration of the intervention. Nevertheless, secondary outcomes suggest some positive effects for the intervention group. However, participation in the intervention group was lower than expected at both the nursing home and resident levels, limiting the validity of the results.

## Introduction

Nursing Home Residents (NHRs) with chronic diseases take a greater number of potentially inappropriate medications, have higher rates of hospitalization and are often hospitalized with potentially avoidable conditions [1–3]. For example, around 21% of NHRs in Germany had at least one hospital visit per quarter, compared to 18.3% of their community-dwelling peers [1]. Other countries (e.g., France, Australia, Ireland and Canada) also report a high proportion of NHRs being hospitalized at least once a year [4–7]. The odds of their visit being unplanned (odds ratio [OR]: 1.51; 95% confidence interval [CI]: 1.47–1.56) or potentially avoidable (OR: 1.21; 95% CI: 1.18–1.26) are also higher. Ouslander and colleagues [3] report that over the half of the hospitalization within the NHR sample in the US context were potentially avoidable. The percentage of avoidable hospitalizations depends on the definition of diagnoses considered to be treatable in ambulatory care and ranges internationally between 4% and 55% [8–10].

A recent German study found that 27% of hospital admissions were potentially avoidable in NHRs [11]. Moreover, NHRs are more likely to be hospitalized for potentially avoidable hospitalizations than non-NH patients [11], resulting in higher costs [3]. Every hospital visit, even for ambulatory care, presents considerable risks and stressors for NHRs [10]. Moreover, NHRs are more likely to develop pressure ulcers, delirium and infections than older people admitted from the community [12]. Adverse clinical consequences are common, and despite intensive use of healthcare resources, mortality remains high [12].

An important driver of avoidable hospitalizations is medication-related issues [13], and polypharmacy is known to be a significant predictor of hospital admissions (risk ratio: 1.79; 95% CI: 1.50–2.12) [14], as are several other iatrogenic diseases [12]. Unplanned hospital admissions can also be caused by difficulties in assessing the severity of a sudden medical issue due to the lack of a clear plan for the management of severe health decline by the responsible nursing and medical staff [15] or poor interprofessional communication, particularly with specialists [16]. Research suggests that maintaining regular communication between general practitioners (GPs) and nursing staff could help reduce unplanned hospitalizations [15]. Systematic, formalized cooperation may provide much-needed guidance to optimize interprofessional communication between physicians and nursing staff [17]. However, this potential is not always translated into improvements in the primary outcomes, as suggested by reviews of early studies [17,18]. Initiatives and reforms therefore continue to promote interventions aimed at reducing unplanned hospital admissions and improving medical care in NHs [19–21].

Healthcare reforms in Germany recommend that NHs form cooperation agreements with local physicians in order to improve medical care in NHs; few, however, make use of the contract possibilities [20]. The literature suggests that these cooperation agreements have either not been realized or have failed due to the lack of cultural and structural conditions. A lack of resources and competences in NHs to cope with the organizational effort required to implement the necessary processes may have compounded the issue [21]. The success of initiatives and their interventions in NHs depends on the level of implementation and engagement across stakeholders [19].

To facilitate the implementation of formalized cooperation between GPs and NHs and to reduce hospitalizations by improving the medical care of geriatric patients in NHs, several interventions have been developed mainly through participatory, research-led approaches [22–24]. Their evaluation, however, shows inconsistent effects in terms of hospitalization, hospital days and healthcare costs [25–27].

The SaarPHIR project (*Saarländische PflegeHeimversorgung Integriert Regelhaft*) developed an interprofessional care concept in NHs in a participatory practice-led approach in order to improve the engagement and the implementation. The aim of this study is to evaluate the effect of the interprofessional care concept in NHs in terms of overall NHR hospitalization (primary outcome) [28].

## Methods

This study is based on the CONSORT statement for reporting cluster-randomized trials [29], the RECORD statement applicable to routinely collected health data [30] and the CHEERS reporting standards for health economic evaluations [31]. The paper reports the results of the effectiveness analysis, with its primary and secondary outcomes including health economic aspects based on health insurance claims data. All other analyses carried out as part of the SaarPHIR study are listed in S1 Table. The SaarPHIR study methods have been described elsewhere [28].

### Study design

A prospective, two-armed cluster-randomized controlled, pragmatic trial with 15 months of follow-up was conducted using health insurance claims data. The six administrative districts of the German federal state of Saarland (Merzig-Wadern, Neunkirchen, RV Saarbrücken, Saarlouis, Saarpfalz-Kreis, St. Wendel) were selected as the level of randomization to avoid spillover effects: GPs could have been contracted by several NHs, and this was less likely to occur across district borders. The facilities were defined as clusters, which were assigned to the intervention (IG) or control group (CG) according to their district. The piloting of SaarPHIR began in April 2018 in Saarbrücken. The cluster-randomized controlled trial (cRCT) was performed from May 2019 until July 2020 (15 months). The implementation of the intervention started at the beginning of the study phase. Delays during recruitment led to a 1-month postponement from the originally scheduled start (April 2019). Additionally, the intervention phase was extended by 3 months to 15 months due to pending recruitments and contractual commitments with physicians as well as pandemic-related issues. The trial was reported in the German Clinical Trials Register (ID: DRKS00017129) before the start of the cRCT. Any deviations from the study protocol, such as changes in timing or post hoc defined outcomes, are tabulated and explained in the S2 Table.

### Study setting and participants

The SaarPHIR study included all NHs in the German federal state of Saarland with at least 50 resident beds and a signed letter of interest (LOI) or, initially, another form of commitment (e.g., verbal agreement) before randomization. Each NH was assigned either to the IG or to the CG.

With the aim of a complete enumeration, residents in NHs were included if they lived in the NH during the cRCT phase, were insured by one of the six participating statutory health insurers (SHIs) and had received a long-term care grade based on their ability to perform activities of daily living. NHRs who lived in more than one NH during the cRCT phase were excluded. The criterion was defined post hoc, as the data set lacked information regarding the time of entry and exit from a NH. This resulted in the inability of the NHR to be assigned to the corresponding NH at the respective time. To be included in the intention-to-treat (ITT) analyses (closed cohort, CC), residents had to live in a participating NH at the beginning of the intervention period. Residents were observed until a change of SHI or until the end of their residential care, which could be due to death or moving to another NH, for example.

### Sample size

Sample size calculation was performed at the NHR level, considering clustering at the NH level rather than the district level. The sample size was therefore calculated based on the level of analysis rather than the level of randomization.

According to Ouslander et al. [3], 32 facilities with 50 residents each for a total of 1,600 residents (assuming equal distribution between study arms) are needed to detect an effect size of 0.6 with a power of 80% and an anticipated between-cluster variance of 0.10 (calculated with R using ClusterPower [32]).

### Randomization

In this study, clustering occurs at two levels: the district level and the NH level. Randomization was performed with a 1:1 randomization algorithm at the district level, rather than at the NH level which would have been the lowest possible level, to avoid contamination. Since hospitalization rates between the regions were similar at baseline, balancing the study arms was not considered in the randomization process. An independent researcher, who was blinded to the districts, received a list with a pseudonym for each district. The researcher computer-generated the randomization sequence, allocated the assignment to pseudonyms and concealed it until the start of the study phase. The Saarland Association of Care was responsible for coding and decoding the district pseudonyms, enrolment of the NHs prior to randomization and the announcement of the intervention allocation to the NHs. The intervention type did not allow for GPs, NHs or NHRs to be blinded to treatment allocation. The researcher conducting the analysis of the outcome parameter was not aware which observations received treatment.

### Intervention and control

The development of the intervention was organized by local groups of healthcare professionals from NHs, GPs and representatives of the statutory health insurances. The intervention involves a concept of interprofessional collaboration in which GPs collectively form regional teams. These medical care teams are encouraged to engage in closer collaboration with NH staff. In addition, the interprofessional care concept included the implementation of a coordinating nurse, extended on-call duty, pre-weekend visits, team meetings, regular screenings and assessments and the development of procedural guidelines to decrease hospitalization rates. Pre-weekend visits should be organized by nurses, and the care teams (consisting of the participating GPs of a region) should be contacted prior to notifying emergency services, if possible. The medical care team should, through shared organization and pooled resources, be able to work more efficiently by coordinating visits. The participating NHs and physicians were provided with financial compensation to facilitate the implementation. The feasibility of the intervention was tested in a pilot study 12 months before the start of the cRCT and is not addressed in this article. As a result of the pilot study, the structural requirements for medical care teams were relaxed slightly, as they made it extremely difficult to recruit physicians and set up the teams. This means, for example, that the requirements for the extended on-call duty were expressed as a recommendation rather than a requirement. The final intervention was described in detail according to the Template for Intervention Description and Replication [33]; see S3 Table. The strategies that supported the implementation of the intervention were summarized according to Proctor et al. [34]; see S4 Table. Further information on the intervention, implementation and piloting are published in the study protocol [28].

NHRs in the CG received usual care: that is, they received medical care from their GP. NH staff did not receive any support in identifying deteriorations in their residents’ state of health, and the communication with the GP was usually limited to their office hours.

### Outcome measures

The primary outcome was hospitalization at the resident level during the cRCT phase from May 2019 to July 2020 (15 months). Each readmission was counted as one hospitalization. First, as well as recurrent, hospitalizations were considered, as the aim of the intervention was to reduce the total number of hospitalizations.

Analyses of secondary outcomes, as stated in the study protocol, included hospitalizations with diagnoses sensitive to improved quality of ambulatory care (ACSCs) according to [35], all-cause deaths during the cRCT phase and hospitalization days. Hospitalizations due to ambulatory-sensitive diagnoses are cases that can be avoided through effective management of chronic diseases, effective acute treatment in the outpatient sector or immunizations. Faisst and Sundmacher [27] have developed a physician-consented list of ambulatory-sensitive diagnoses for the German outpatient sector with no special focus on NHRs.

During data analysis, a more specific list of diagnoses with a focus on NHRs was published, called NH-sensitive diagnoses (NHSCs) [36]. NHSCs are cases that can be avoided if NHRs receive optimal medical care. The list was developed in two steps first via claims data and secondly by experts in a Delphi process [36]. Furthermore, they published an expert-consented list of six elements and interventions to avoid NHSCs and to improve the healthcare of NHRs. The list includes elements such as collaboration and communication as well as internal processes in NHs for improved interaction with collaborators [37], which were also represented in the interprofessional care intervention of the SaarPHIR project. Due to the specific focus on the medical care of NHRs and the overlap between the authors’ recommendations and the intervention studied in the SaarPHIR project, this outcome was included post hoc as an additional secondary outcome. Health economic analyses considered the overall costs that were covered by the SHI during the study phase and the costs per sector, including, for example, ambulatory and dental costs. All outcomes were observed at the resident level.

### Data collection

All analyses were based on health insurance claims data. A project-specific data security plan had been established to ensure confidentiality of all collected data in adherence to European and German laws and the Good Clinical Practice guideline [38]. Claims data transfer was authorized according to Section 75 of the German Social Code Book X (Sozialgesetzbuch X, §75 SGB X) by the state offices of Saarland, Lower Saxony and Rhineland-Palatinate for AOK and the Federal Office for Social Security and the Saarland Association of Statutory Health Insurance Physicians (German: *Kassenärztliche Vereinigung Saarland*) for the other five participating SHIs (BARMER, Techniker Krankenkasse, Betriebskrankenkassen, DAK-Gesundheit, Knappschaft-Bahn-See). These claims data were provided from April 2018 until April 2021 by the participating insurers and the Association of Statutory Health Insurance Physicians and were the main source for primary and secondary endpoints. Residents and their respective NHs were identified by the SHIs on the basis of the inclusion criteria, i.e., residents living in a NH, who were insured by one of the six participating SHIs and had a long-term care grade during the data delivery period. In a second step, an independent researcher selected residents who received inpatient care services during the cRCT phase.

An external data trustee supervised the data pseudonymization, which was applied to identifying data (insured persons’ names, NHs, physicians and physicians’ practices) and executed in a three-step process. First, data suppliers used an agreed-upon pseudonymization process and transferred data to the data trustee, who generated project pseudonyms. These data were transferred to evaluating researchers, who generated study pseudonyms. The data trustee also received a list of NHs that had signed a LOI, including their institution number, which was pseudonymized according to the agreed process. The pseudonymized list was forwarded to the researcher who conducted the analyses and was used to identify the participating NHs and their residents. The data of the insurers and the Association of Statutory Health Insurance Physicians were linked by the created pseudonyms. Data access was granted only to researchers involved in the evaluation. After the study is completed, the data will be archived for 10 years.

### Statistical analyses

#### Effectiveness analyses.

The study population was characterized inter alia by the Health-Related Quality of Life Composite Index [39], the Charlson Comorbidity Score [40] and the Diederichs List of Chronic Diseases [41] to adequately describe comorbidities based on validated International Statistical Classification of Diseases and Related Health Problems codes. The primary statistical analysis was an individual-level ITT analysis that accounted for clustering at the NH level, using hospitalization (including recurrent events) as a dependent variable in a generalized linear mixed model assuming a Poisson distribution, with resident’s time at risk for hospitalization as the denominator. Residents were included if they lived in a participating NH at the start of the cRCT phase (May 2019) and were insured with one SHI; that is, changing insurance providers or NH ended the observation for a person, as did death. Since the intervention was randomly assigned at the district level, the primary analysis considered the NH level as random effect, intervention allocation and the number of hospitalizations 12 months prior to the cRCT start as fixed effects. Under normal circumstances, the randomized cluster level would be adopted for analysis. To avoid a contamination bias, randomization was performed at district level. This means that only six clusters were available for randomization. However, a minimum of eight clusters is recommended for the analysis of a cRCT [42]. Thus, the sample size calculation and the analyses were conducted at the individual level and considered the NH as a cluster level to ensure a pragmatic evaluation of the intervention within the complex healthcare system. This unconventional approach assumed, that the NHs were characterized by the districts in which they operate. To test this assumption, post hoc sensitivity analyses were conducted with primary and secondary outcomes that included district as a random effect and NHs as a nested random effect within districts. Age, sex and comorbidity (i.e., Charlson Comorbidity Score) were included as fixed effects in the sensitivity analyses to account for potential differences. In addition to the Poisson regression, a negative binomial regression was carried out as part of the sensitivity analyses to determine the overdispersion typical for hospitalizations.

The same models were used in the sensitivity analyses of per-protocol (PP) populations. Within the analysis plan, two options were defined as PP populations: (1) the subgroup of NHs that actively participated in the IG according to the study protocol, while the CG participants remained the same (NH-PP); and (2) a subgroup of the first that included IG residents only if they received project-specific services (R-PP). Residents receiving project-specific services were identified by project-specific billing codes in the SHI data. PP populations were defined as either open or closed cohort. The open cohort included residents who lived in the NH either at the beginning of the study or at any point during the intervention (NH-PP-OC and R-PP-OC). The closed cohort included residents who lived in the NH at the beginning of the study (NH-PP-CC and R-PP-CC). The analysis populations mentioned are listed and described in detail in Table 1.

**Table 1:** Description of the analyses populations.

Analysis population	Abbreviation	Description
Intention to treat population	ITT	To be included in the intention-to-treat analyses, residents had to live in a participating NH at the beginning of the intervention period. This meant that, by definition, the analysis was carried out with a closed cohort.
Per protocol population	PP	Within the analysis plan, two options were defined as per protocol populations:
Per protocol populations defined at NH level	NH-PP	The subgroup of NHs that actively participated in the IG according to the study protocol, while the CG residents remained the same. The subgroup was defined as both an open and a closed cohort per protocol population at NH level.
Per protocol populations defined at residents’ level	R-PP	The subgroup of the NH-PP that included IG residents only if they received project-specific services and CG residents if they did not. The subgroup was defined as both an open and a closed cohort per protocol population at resident level.
Per protocol populations defined at NH level in an open cohort design	NH-PP-OC	The population was defined as an open cohort per protocol population at NH level, including 1) NHs that actively participated in the IG according to the study protocol, while the CG residents remained the same and 2) all residents who lived in a participating NH at any time during the study period.
Per protocol populations defined at residents’ level in an open cohort design	R-PP-OC	The population was defined as an open cohort per protocol population at resident level, including only 1) IG residents if they received project-specific services and CG residents if they did not and 2) all residents who lived in a participating NH at any time during the study period.
Per protocol populations defined at NH level in a closed open cohort design	NH-PP-CC	The population was defined as an open cohort per protocol population at NH level, including 1) NHs that actively participated in the IG according to the study protocol, while the CG residents remained the same and 2) only residents who lived in a participating NH at the beginning of the study period.
Per protocol populations defined at residents’ level in a closed cohort design	R-PP-CC	The population was defined as an open cohort per protocol population at resident level, including only 1) IG residents if they received project-specific services and CG residents if they did not and 2) only residents who lived in a participating NH at the beginning of the study period.

All secondary outcomes were analysed using generalized linear mixed models assuming a Poisson distribution for count variables and normal distribution for dichotomous variables. Study group, hospitalizations 12 months prior to intervention, age, gender and comorbidity [40] were defined as fixed effects and NH membership as a random effect for the cRCT phase, unless noted otherwise. The analyses of the secondary endpoints were based on the NH-PP-OC to assess the efficacy potential of the interprofessional care concept.

#### Health economic analyses.

As part of the health economic evaluation, costs were analysed descriptively and exploratively starting in July 2019. This was done with regard to the NH-PP-OC population, which was selected due to its liberal character to assess the efficacy potential. Ambulatory costs were considered in a quarterly structure, as they cannot be allocated to smaller time increments. Consequently, observations were made on a person-quarter basis. Since the intervention started in May 2019, cost analyses did not align perfectly with the cRCT phase. This led to a reduction of the underlying population, since NHRs who died in the second quarter of 2019 were excluded. In order to adequately describe the sub-population of interest, their characteristics have been summarized descriptively for each study group. Residents’ costs were considered if they were observed for an entire quarter. Ambulatory and dentist costs were already available on a quarterly basis. Inpatient costs were attributed to the quarter the resident was discharged in, medications and medical aids to the day they were handed over. Costs for remedies and rehabilitations were distributed to quarters proportionally. Costs for each resident for every quarter were added up to total costs and used as the dependent variable. Costs for each sector were analysed separately and corrected for zero inflation using two-part regression models: first, a logistic model adjusted for zero inflation and then a generalized linear conditional model with a log-link and a modified gamma distribution was performed to estimate regression coefficients [43]. The average costs per resident in the 12 months prior to July 2019, age and comorbidity [40] were included as independent variables. Calendar time was included to adjust for time trends. The NH and the NHR level were considered as random effects.

In all analyses, a 5% significance level was used, and cluster-adjusted 95% confidence intervals were calculated for each model. Analyses were performed with the free software R (the R Foundation for Statistical Computing) using the packages “gtsummary”, “lme4” and “glmmTMB” [43–45].

### Data access and cleaning methods

Each insurer provided data on the costs they are legally required to reimburse (i.e., ambulatory and inpatient care, pharmaceuticals, remedies, therapeutic appliances, medical aids, transport, rehabilitation, dental care) and administrative and medical information (e.g., hospitalization dates and diagnoses) based on the described inclusion criteria. Further data selection and cleaning were performed by an independent researcher. In the subsequent selection process, NHs with a LOI were identified using a pseudonymized institution number. The Association of Statutory Health Insurance Physicians provided data on project-specific services, which were linked by an independent researcher using the pseudonyms at the resident level. Plausibility analyses were conducted descriptively, information on gender and age was compared to data from the Federal Statistical Office and gender-specific diagnoses were verified. If necessary, insurers were asked to revise incorrect information. Some hospital admission dates overlapped, resulting in some residents being admitted twice before being discharged. Such cases were recoded as one hospital admission when the separate entries were due to the data coding of the insurers. The coded diagnoses based on the International Statistical Classification of Diseases and Related Health Problems were validated for further analyses according to Schubert et al. [46].

### Ethical and legal considerations

Approval for this study was granted by the Ethics Committee of the Saarland Medical Association on March 4th, 2019 (No. 56/18). For the claims data of the SHIs, permission to transfer data for research purposes in accordance with §75 SGB X, was also obtained from the responsible supervisory authorities, the Federal Social Office (Bundesamt für Arbeitsschutz und Sozialordnung, BAS). Approval was granted on the basis of a data protection concept developed in advance to ensure that the processing of secondary data was handled securely in accordance with data protection law. NHRs did not provide informed consent for the transfer and analysis of the pseudonymized claims data, as the claims data-based endpoints were to be analysed for all residents of the participating NHs in the six districts of Saarland in order to be able to fully evaluate the overall effectiveness of the intervention. The transfer of social data without informed consent was approved by the BAS on the basis of §75 SGB X. However, all actively participating residents, i.e., residents receiving project-specific services, provided informed consent prior to their involvement in the study or could opt-out.

## Results

### Participants and baseline characteristics

**Fig 1:**
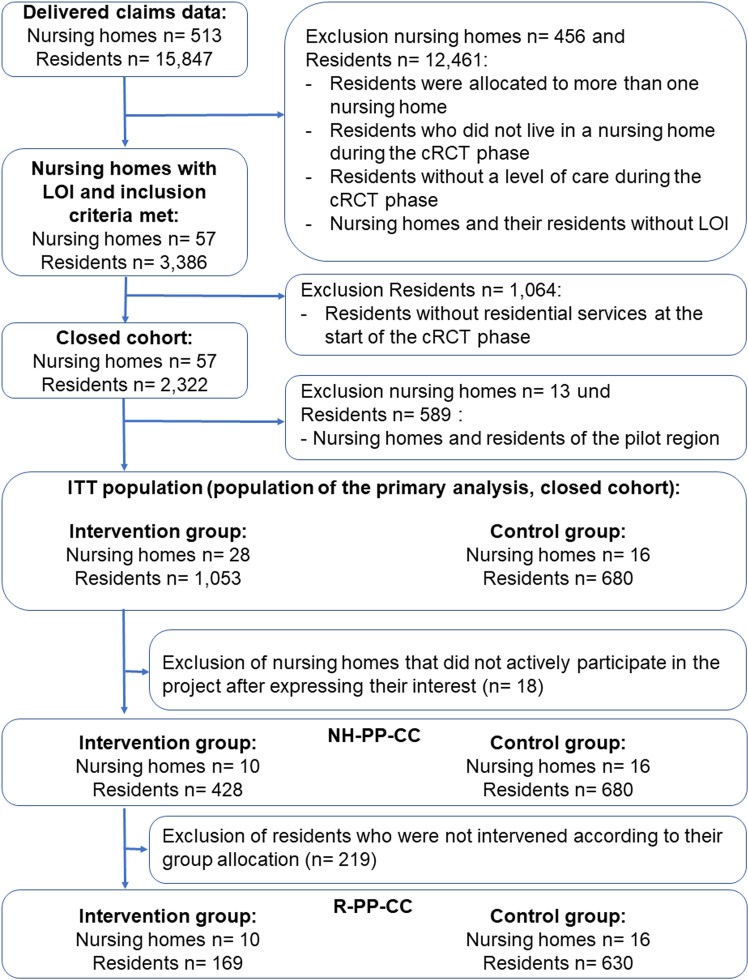
Flowchart of the claims data provided and in-/exclusion of insured residents in the closed analyses populations. Abbreviations: cRCT = Cluster-randomized controlled trial, LOI = Letter of interest, NH-PP-CC = Nursing home per protocol closed cohort (subgroup of NH that were actively participating in the intervention group according to the study protocol, while the control group participants remained the same), R-PP-CC = Residents per protocol closed cohort (subgroup of NH-PP-CC including intervention group residents only if they received project-specific services).

Six districts were randomized in which 44 nursing homes signed an LOI (see S5 Table). The six participating SHIs supplied data on 15,847 NHRs with a long-term care grade living in a NH in Saarland during the data delivery period, from which the NHRs of the six randomized districts and the 44 NHs with LOI were filtered. Thus, 14,114 NHRs were excluded because they did not live in a NH during the cRCT phase, could not be assigned to a NH with a signed LOI or were assigned to a NH participating in the pilot phase. In addition, an exclusion criterion was established post hoc, pertaining to a limited number of the 14,114 NHR, specifically those residing in more than one NH during the cRCT phase. Finally, 1,733 residents from 44 NHs were screened eligible and allocated to the IG (NHRs: 1,053; NHs: 28) and CG (NHRs: 680; NHs: 16) according to the NH’s location (see Fig 1). These 1,733 residents constituted the ITT population, where the majority of the 44 NHs expressed their interest in participating in the study before randomization (39 out of 44 nursing homes). As the sample size was not reached, recruitment continued after the start of the study and five NHs expressed their interest after randomization and subsequently joined the study. Of the 28 NHs in the IG, 10 implemented the intervention during the cRCT and thus formed the IG of the NH-PP-CC population, including 428 NHRs. The CG of the NH-PP-CC population is by definition the same as the CG of the ITT population. In the R-PP-CC population, 169 NHRs of the IG received project-specific services, while 630 NHRs of the CG did not. However, 50 NHRs of the CG received project-specific services and were excluded from the R-PP-CC population. A separate flowchart for the open cohort populations is provided in S1 Fig.

The NHRs of the six randomized districts had an average age between 80 and 84 years. The average number of hospitalizations in the districts prior to the cRCT was comparable between study groups (see S5 Table). Furthermore, the person-years observed did not differ between IG and CG (see S5 Table). Descriptive characteristics of the ITT population were similar to the reference population of the federal state of Saarland and revealed no significant differences in baseline characteristics between the IG and CG. At baseline, residents mean age was 83 years (*SD* = 10) in the IG, 80 years (*SD* = 11) in the CG; 72% (*n* = 620) of the IG was female, compared to 65% (*n* = 398) in the CG. The characteristics per district did not differ between the groups as displayed in S5 Table. The distribution of long-term care grade was similar, as was the number of hospitalizations (see Table 2). The intra-cluster correlation coefficient for the hospitalization 12 months before the cRCT start, if one defined hospitalization as a dichotomous variable, was 0.032 overall and 0.029 versus 0.048 in the IG and CG, respectively.

**Table 2:** Baseline characteristics of the intention-to-treat population by randomized group.

	Overall *N* = 1,733	IG *n* = 1,053	CG *n* = 680
**Age at baseline in years [mean (*SD*)]**	82 (11)	83 (10)	80 (11)
**Sex, female [***n* **(%)]**	1,179 (68%)	739 (70%)	440 (65%)
**Long-term care grade**^1^ **[***n* **(%)]**			
1 (minimal care need)	48 (2.8%)	23 (2.2%)	25 (3.7%)
2 (substantial care need)	542 (31.3%)	335 (31.8%)	207 (30.4%)
3 (severe need)	606 (35.0%)	363 (34.5%)	243 (35.7%)
4 (very severe need)	384 (22.2%)	232 (22.0%)	152 (22.4%)
5 (very severe need with special requirements for nursing care)	153 (8.8%)	100 (9.5%)	53 (7.8%)
**Health-Related Quality of Life Composite Index [median [1st quartile; 3rd quartile]]**	11 [7;15]	11 [7;1 5]	11 [7;16]
**Charlson Comorbidity-Index [median [1st quartile; 3rd quartile]]**	3 [1;5]	3 [1;5]	3 [1;5]
**Diederichs List of Chronic Cis. [median [1st quartile; 3rd quartile]]**	4 [3;6]	4 [3;6]	4 [2;6]
**Average no. of hospitalizations before cRCT**	1.01 [1.39]	1.00 [1.36]	1.04 [1.44]

^1^Individuals undergo an assessment and evaluation process to determine their level of care needs. Based on this assessment, they are assigned a specific care grade that corresponds to the benefits provided through long-term care insurance. The five care grades are designed to account for the individual’s capacity to manage persistent physical, cognitive or psychological impairments, as well as any health-related stresses or requirements they may have.

^2^Due to technical constraints, comorbidity scores were missing for 58 NHRs. Abbreviations: CI = Confidence interval, CG = Control group, IG = Intervention group.

Given the potential impact of the COVID-19 pandemic on the study, the incidence of COVID-19 cases during the study period was determined and found to be approximately 7% in both groups in the closed ITT population (see S6 Table).

### Effectiveness analysis

**Fig 2:**
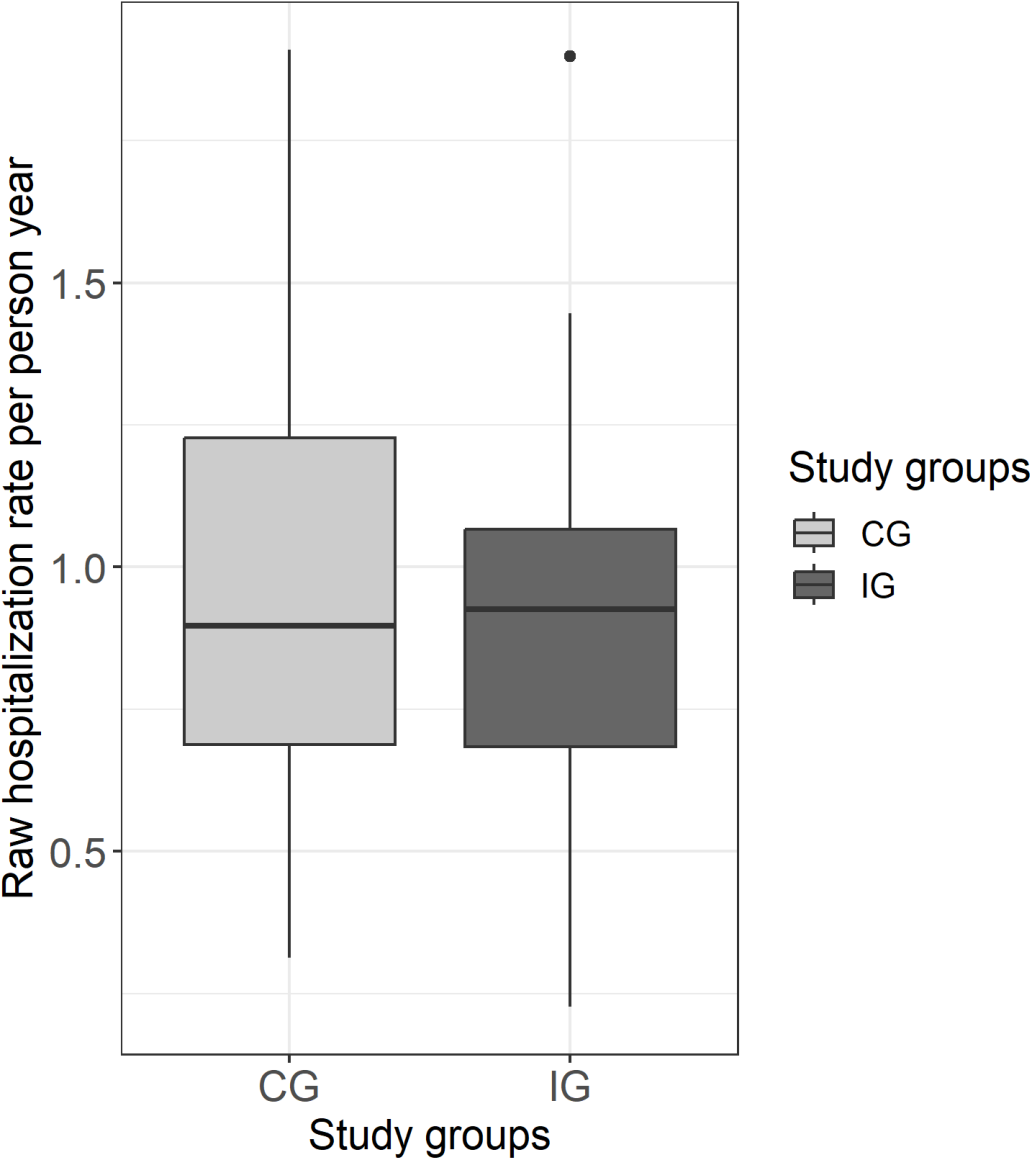
Boxplot of observed (crude) hospitalizations per person-year in the cRCT phase per study group. Abbreviations: CG = Control group, IG = Intervention group.

The median crude hospitalization rate per person-year was just over 1 in both groups and thus shows no substantial difference between the study groups. The crude rate indicates a certain variation in both groups, with the interquartile range being slightly higher in the CG (see Fig 2). Neither the primary analysis nor the sensitivity analyses in the ITT or NH-PP-CC population (Models 1–4) show a significant effect of the intervention on the hospitalization rate (Table 3). Including the district level as a random effect did not change the results (see S7 Table). Overall, women were less likely to be hospitalized than men (incidence rate ratio [IRR]: 0.74, 95% CI: 0.66–0.82), as were people with lower Charlson Comorbidity Scores (IRR: 1.06, 95% CI: 1.04–1.08) (see Table 3, Model 3). Sensitivity analyses with the R-PP-CC population showed that the intervention can affect the hospitalization rate (IRR: 0.69; 95% CI: 0.52–0.93), after adjusting for age, sex and comorbidity (see Table 3, Model 5).

**Table 3:** Results for the primary endpoint – effects on the hospitalization rate in the ITT and R-PP-CC population.

	Model 1 NH: N = 44 NHR: N = 1,733			Model 2 NH: N = 44 NHR: N = 1,733			Model 3 NH: N = 44 NHR: N = 1,675^1^			Model 4 NH: N = 26 NHR: N = 1,071^1^			Model 5 NH: N = 26 NHR: N = 771^1^		
Hospitalization	IRR	95% CI	p-value	IRR	95% CI	p-value	IRR	95% CI	p-value	IRR	95% CI	p-value	IRR	95% CI	p-value
**(Intercept)**	0.00	0.00, 0.00	<0.001	0.00	0.00, 0.00	<0.001	0.00	0.00, 0.00	<0.001	0.00	0.00; 0.00	<0.001	0.00	0.00; 0.00	<0.001
**CG**	–	–		–	–		–	–		–	–		–	–	
**IG**	0.94	0.78; 1.14	0.5	0.97	0.81; 1.16	0.7	0.96	0.80; 1.16	0.7	0.90	0.74; 1.10	0.3	0.69	0.52; 0.93	0.015
**Hospitalization** **before cRCT**	1.31	1.27; 1.34	<0.001	1.42	1.35; 1.49	<0.001	1.27	1.23; 1.30	<0.001	1.5	1.21; 1.29	<0.001	1.24	1.20, 1.29	<0.001
**Sex**															
**Male**							–	–		–	–		–	–	
**Female**							0.74	0.66; 0.82	<0.001	0.74	0.65; 0.85	<0.001	0.88	0.75; 1.03	0.10
**Age**							1.00	1.00; 1.01	0.15	1.01	1.00; 1.02	0.003	1.01	1.00; 1.02	0.007
**Charlson** **comorbidity index**							1.06	1.04; 1.08	<0.001	1.05	1.03; 1.08	<0001	1.05	1.02; 1.07	0.001
**Random effect NH** Intercept *SD*	0.25			0.15			0.24			0.19			0.27		

^1^ Please note that the number of residents included in these models differs from the number of residents in the respective populations, as it was not possible to calculate a comorbidity score for all residents. Abbreviations: IRR = Incidence rate ratio, CI = Confidence interval, CG = Control group, IG = Intervention group, cRCT = Cluster-randomized controlled trial, SD = Standard deviation, ITT = Intention to treat. The primary endpoint includes all hospitalizations during the observation period (including incident and recurrent hospitalizations). Model 1: results of the primary analysis; IRR and CI were estimated using Poisson regression in the ITT population in the primary observation period. Model 2: in contrast to Model 1, IRR and CI were estimated using negative binomial regression. Model 3: in contrast to Model 1, further independent variables were included in the analysis as fixed effects. Model 4: in contrast to Model 1, the first per-protocol population (NH-PP-CC) was considered in the analysis, i.e., only NHs that actively participated in the IG according to the study protocol. Model 5: in contrast to Model 1, the second per-protocol population (R-PP-CC) was considered in the analysis, i.e., only residents who also received an intervention service were included in the IG.

**Fig 3:**
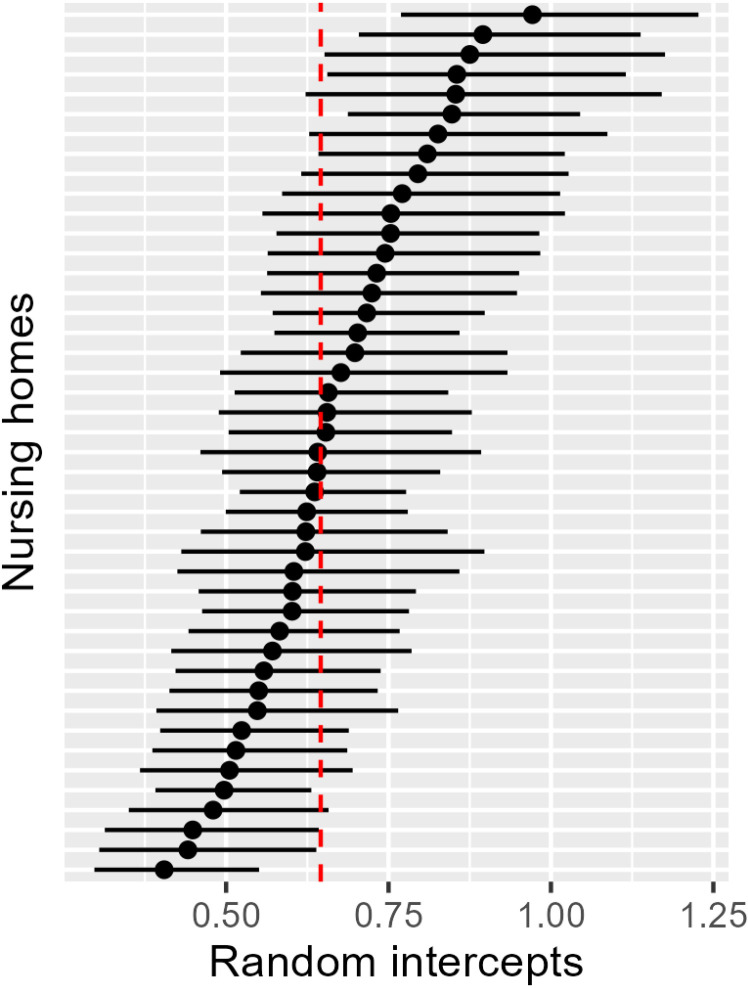
Estimated base hospitalization rates per person-year (random intercept) including 95% confidence interval per nursing home and the estimated mean base hospitalization rate per person-year (red dashed line, fixed intercept) based on the primary analysis model.

The standard deviation of the random effects at the NH level is largely identical for Models 1, 3 and 5 at approximately 0.24 and decreased to 0.15 for Model 2 (see Table 3). The lower standard deviation of the random effect in Model 2 can be explained by the negative binomial regression and the associated correction for overdispersion. Fig 3 shows the intercepts of the NHs including 95% confidence intervals to illustrate the dispersion of the estimated baseline hospitalization rates per person-year (adjusted for hospitalization before cRCT). NHs are represented with approximately 0.4 hospitalizations per person-year and up to approximately 1.0 hospitalizations per person-year. This variation was accounted for by the multilevel model.

Table 4 shows the results of the secondary outcomes, where the NHs of the IG in the NH-PP-OC population had significantly fewer hospitalizations due to NHSCs (IRR: 0.75; 95% CI: 0.59–0.96) and showed positive tendencies in reducing their hospitalizations due to ACSCs (IRR: 0.85; 95% CI:0.66; 1.09). The intervention had no impact on the mortality rate or the total days of hospitalizations (see Table 4). Including the district level as a random effect did not change the results (see S8 Table).

**Table 4:** Regression results of the secondary outcomes in the NH-PP-OC population.

Outcomes	ACSC NH: N = 26 NHR: N = 1,566^1^			NHSC NH: N = 26 NHR: N = 1,566^1^			Mortality NH: N = 26 NHR: N = 1,566^1^			Hospital days NH: N = 26 NHR: N = 1,566^1^		
	IRR	95% CI	p-value	IRR	95% CI	p-value	MRR	95% CI	p-value	Effect	95% CI	p-value
**(Intercept)**	0.00	0.00, 0.00	<0.001	0.00	0.00, 0.00	<0.001	0.00	0.00, 0.00	<0.001	5.3	3.5; 7.1	<0.001
**CG**	—	—		—	—		—	—		—	—	
**IG**	0.85	0.66; 1.09	0.2	0.75	0.59; 0.96	0.021	1.05	0.78, 1.41	0.8	−0.26	−2.1; 1.5	0.8
**Hospitalization** **before cRCT**	1.23	1.19; 1.27	<0.001	1.33	1.25; 1.41	<0.001	1.10	1.04, 1.15	<0.001	1.6	1.2; 2.1	<0.001
**Sex**												
**Male**	–	–		–	–		–	–		–	–	
**Female**	0.73	0.61; 0.88	<0.001	0.73	0.58; 0.91	0.005	0.61	0.50, 0.74	<0.001	−1.1	−2.6; 0.41	0.2
**Age**	1.01	1.00; 1.02	0.013	1.02	1.01; 1.03	0.003	1.06	1.05, 1.07	<0.001	−0.07	−0.14; 0.00	0.048
**Charlson** **comorbidity index**	1.09	1.06; 1.12	<0.001	1.07	1.03; 1.11	<0.001	1.04	1.01, 1.08	0.018	0.19	−0.05; 0.44	0.13
**Random effect NH** Intercept *SD*	0.22			0.14			0.28			1.1		
Residual *SD*										14		

^1^Please note that the number of residents included in these models differs from the number of residents in the respective populations, as it was not possible to calculate a comorbidity score for all residents. Abbreviations: ACSC = Ambulatory care-sensitive conditions, NHSC = Nursing home-sensitive conditions, IRR = Incidence rate ratio, MRR = Mortality rate ratio, CI = Confidence interval, CG = Control group, IG = Intervention group, cRCT = Cluster-randomized controlled trial, *SD* = Standard deviation, ITT = Intention to treat. Model ACSC: includes all (incident and recurrent) hospitalizations due to ACSCs during the cRCT; IRR and CI were estimated using Poisson regression in the NH-PP-OC population. Model NHSC: in contrast to Model 1, hospitalizations due to NHSCs were included; IRR and CI were estimated using negative binomial regression. Model Mortality: the analysis included death from all causes which occurred during the cRCT; MRR and CI were estimated using Poisson regression in the NH-PP-OC population. Model Hospital days: days spent in hospital during the cRCT; effect and CI were estimated using a linear regression in the NH-PP-OC population.

### Health economic analyses

As mentioned above, in the IG, 10 NHs implemented the intervention during the cRCT. Overall, 221 NHRs in the IG received project-specific services, while NHRs in the CG did not (see S1 Fig). The health economic analyses included data from 1,312 NHRs with a total of 4,327 person-quarters. Descriptive characteristics of the population considered were similar to those of the ITT population of the primary analysis, and there were no significant differences in the observed characteristics between the IG and CG, as Table 5 shows.

**Table 5:** Baseline characteristics of the population used for the health economic evaluation by randomized group.

	Overall *N* = 1,312	IG *n* = 516	CG *n* = 796
**Age**	81 (11)	82 (11)	80 (11)
**Sex, female [***n* **(%)]**	859 (65%)	352 (68%)	507 (64%)
**Long-term care grade**^1^ **[***n* **(%)]**			
1 (minimal care need)	40 (3.0%)	15 (2.9%)	25 (3.1%)
2 (substantial care need)	409 (31%)	164 (32%)	245 (31%)
3 (severe need)	482 (37%)	186 (36%)	296 (37%)
4 (very severe need)	286 (22%)	114 (22%)	172 (22%)
5 (very severe need with special requirements for nursing care)	95 (7.2%)	37 (7.2%)	58 (7.3%)
**Charlson Comorbidity Index [median [1st quartile; 3rd quartile]]**	3 [1;5]^3^	3 [1;5]	3 [1;5]
**Costs (€) 12 months before cRCT [mean (*SD*)]**	3,068.52 (3,798.94)	3,254.10 (4,292.39)	2,952.13 (3,449.49)
**Number of resident quarters observed**^2^ **[***n***]**	4,237	1,633	2,604

^1^Individuals undergo an assessment and evaluation process to determine their level of care needs. Based on this assessment, they are assigned a specific care grade that corresponds to the benefits provided through long-term care insurance. The five care grades are designed to account for the individual’s capacity to manage persistent physical, cognitive or psychological impairments, as well as any health-related stresses or requirements they may have.

^2^Refers to the quarterly data structure observed in the SHI claims data and serves as the reference population in the analyses.

^3^Due to technical constraints, comorbidity scores were missing for 45 NHRs.

The crude difference in total costs between groups was €20.06 per person-quarter. The regression results for each sector and total costs can be seen in Table 6. The analysis showed that the intervention was not more expensive than routine care (€59.40; 95% CI: −178.20–356.40), but it did increase medication costs significantly (€82.53; 95% CI: 11.79–165.06), and costs for outpatient physician services provided by hospitals were reduced (−€40.80; 95% CI: −76.50–0.00).

**Table 6:** Absolute intervention effects based on the regression analyses with reference to the NH-PP-OC population.

	Effects^1^ NH: N = 26 NHR: N = 1,267^1^	95% CI	p-value
Hospital (inpatient) services			
Number of hospital stays	−0.06	−0.17; 0.06	0.30
Number of hospital days	−0.26	−2.10; 1.50	0.8
Hospital costs (€)	125.19	−417.30; 792.87	0.70
Ambulant (outpatient) service costs (€)	0.00	−67.36; 79.99	>0.9
Ambulant (outpatient) services costs in hospitals (€)	−40.80	−76.50; 0.00	0.053
Medication costs (€)	82.53	11.79; 165.06	0.017
Outpatient non-physician services costs (€)	−5.60	−53.20; 54.91	0.8
Transport costs (€)	−98.56	−211.20; 42.24	0.15
Rehab services costs (€)	−896.96	−1,709.83; 476.51	0.2
Dental services costs (€)	8.75	−30.00; 65.00	0.7
Total costs (€)	59.40	−178.20; 356.40	0.6

^1^Values can be interpreted as absolute intervention effects in relation to the average population characteristics (i.e., reduced or increased service utilization per resident [for costs per quarter, for stays per person-year]). Results are based on the NH-PP-OC population between quarter 3 in 2019 and quarter 2 in 2020 (or in the cRCT-phase) in comparison between IG and CG. Consequently, NHRs censored at the end of the second quarter of 2019 were not observed, which resulted in a reduction of the NH-PP-OC population. Further differences to the NH-PP-OC population are attributable to missing comorbidity scores for a small number of NHRs.

^a^Data per resident and person-year.

^b^Data per resident in the primary observation period; all other values per resident per quarter.

As shown in the intervention-specific fees in Table 7, the SaarPHIR care fee per capita (German: *Betreuungspauschale*) was billed most frequently, while on-call duty accounted for the largest share of billed costs by physicians.

**Table 7:** Project-specific services of the NH-PP-OC population during the cRCT phase.

Service description (fee number)	Frequency	Proportion (%)	Sum of the billed services (€)
Care fee per capita SaarPHIR (98710)	615	35.06	18,450
On-call duty SaarPHIR (98715)	562	32.04	44,960
Drug therapy safety examination SaarPHIR (98713)	225	12.83	9,000
Pre-weekend visits/ On-site meetings SaarPHIR (98716)	161	9.18	12,880
Extended geriatric assessment SaarPHIR (98711)	115	6.56	690
Team meetings SaarPHIR (98717)	38	2.17	3,800
Drug therapy safety examination after medication change SaarPHIR (98714)	34	1.94	680
Telephone consultation by a specialist (physician) SaarPHIR (98712)	4	0.23	40

## Discussion

### Key results

The interprofessional care concept provided by GPs and NHs did not significantly affect the overall hospitalization rate during the 15 months of the cRCT. A randomized controlled study recently published by Mazur et al. for the German healthcare context, evaluating an intervention focusing on GP–nurse collaboration in NHs similar to the SaarPHIR project, used the proportion of hospitalizations and did not find significant results within 12 months from randomization [25]. However, the literature suggests a potential for reducing avoidable hospitalizations [18,26,47,48]. In a study with a similar intervention to the SaarPHIR project but evaluated in a non-randomized controlled design, participating healthcare professionals reported benefits for their daily work [49]. In addition, they observed a reduction of hospitalizations by 0.08 (p = 0.001) per resident and observed quarter [26] and a better 3-year overall survival [50]. Kada et al. state that 40% of the ambulatory treatments the authors analysed in the emergency department could be classified as avoidable [48]. These findings are reflected in the results of our sensitivity analyses, in which comparable populations and more specific outcomes were employed. For instance, analyses reducing the population to residents who were treated as intended (R-PP-CC) identified positive effects in favour of the intervention (IRR: 0.75; 95% CI: 0.59–0.96).

Overall, no effect was detected in the primary analysis of the intervention. In part, this may have been due to a lack of power, since only 10 NHs implemented the intervention, whereas a minimum of 16 NHs with 50 NHRs each per group were needed to detect a moderate effect. It remains to be investigated, however, whether the overall hospitalization as a patient relevant outcome can be reduced by an interprofessional care concept like it was tested in the SaarPHIR project. In total, 169 of 428 NHRs (61%) of the ITT population received project-specific services, indicating that not all NHRs eligible for the new form of care were provided with it. This could have been for various reasons: for example, because the NHs did not form a medical care team with a GP or because the NHRs or their legal guardian did not respond to the request to participate in a study. Some NHs only decided to implement the intervention after the start of the cRCT. As a result, they had less time, for instance, to form medical care teams and to enrol NHRs into the new form of care, resulting in poor permeation [51]. The results of the process evaluation show that an initial effort was perceived in adapting the daily routine [52]. In addition, a large part of the implementation processes of the interprofessional care concept took place from May 2019 to July 2020, which was during the COVID-19 pandemic, and may have been affected by it. In the publication of the process evaluation, however, the effects of the COVID-19 pandemic played a relatively minor role, being mainly noticeable in the implementation of the evaluation. Overall, the intervention was implemented as planned in the surveyed NHs, although the extent of implementation varied. These aspects are part of the process evaluation, which are published elsewhere [52].

Analyses of secondary outcomes showed that increasing the specificity of the outcome leads to a positive tendency of the intervention, with hospitalization due to ACSCs being reduced for the NH-PP-OC population. Moreover, NHRs in the IG were significantly less likely to be hospitalized due to NHSCs. The reduction of NHSCs appears to be an achievable endpoint, while the total number of all-cause hospitalizations could only be partially influenced by the project. One reason for this could be that the actions recommended by the authors of the NHSC list to reduce NHSCs were included in the interprofessional care concept of this study [37]. Given that the effect of the post hoc added secondary outcome is not reflected in the primary outcome, it may be reasonably assumed that the proportion of hospitalizations due to NHSCs that can be avoided through the intervention is a relatively small proportion of all-cause hospitalizations. This raises the question of whether the intervention was appropriate only for a subpopulation or whether, due to difficulties in recruitment, only a subpopulation received the intervention.

As in studies with a similar design, the interprofessional care concept investigated in this study did not affect the mortality rate [53,54], or the number of days spent in hospitals or costs [53]. Health economic analyses did not include a cost-effectiveness analysis and incremental cost-effectiveness ratios were not applied because the intervention did not show a statistically positive effect. The explorative analyses of costs for the NH-PP-OC show that the intervention was not more expensive than routine care, but it did increase medication costs significantly, although costs for outpatient physician services provided by hospitals were reduced.

The analyses of secondary outcomes, sensitivity analyses and health economic analyses were not powered and employed the open cohort per-protocol population, which is a subpopulation. Hospitalizations due to NHSCs were included post hoc in the analysis plan and analysed as a secondary endpoint. The results are therefore exploratory by nature, and they may potentially be biased and have reduced validity.

## Implications

The evaluated interprofessional care concept did not have an effect on overall hospitalization, but appears to have a positive influence on patient-relevant outcomes and should be investigated further. The SHIs decided against extending project-specific contracts and continuing billing items, effectively ending the intervention. The Federal Joint Committee also ruled against the transfer of the intervention to routine care [55].

## Limitations

The randomization was performed at the district level, rather than the lowest possible level [42] to avoid contamination, since the intervention included regional teams of physicians and NH. However, the analyses were carried out at the individual level and considered the NH as a cluster, as the NHs were assumed to be characterized by the districts in which they operate. To test whether accounting for the district level had an effect on the results, sensitivity analyses were conducted with the primary and secondary outcomes including district as a random effect and NHs as a nested random effect within districts. The results remain the same (see S7 Table and S8 Table). The comparability of the districts and their NHs may be limited, as the randomization process did not consider the balancing of the study arms. The IG included more NHs than the CG and the age was slightly higher in the IG. However, the average number of hospitalizations prior to cRCT was comparable between groups (see S5 Table). The person-years observed in each study arm did not differ as a result of censoring events (see S5 Table). The NHs were recruited prior to randomization in order to avoid bias. The majority of NHs expressed their interest in participating in the study before randomization (39 out of 52 nursing homes). Only five homes expressed interest after the start of the study and thus after randomization. We believe that a significant bias was avoided by prior recruitment. However, recruitment is a challenge in any study, and recruitment after randomization is sometimes unavoidable to ensure the conduct of the study. A considerable proportion of residents in the IG did not receive project-specific services, while several residents in the CG did (n= 50). This could introduce a potential contamination bias. The potential for bias was addressed through the conduction of an ITT analysis. For the analyses of secondary outcomes, the ITT population is typically used for comparison purposes. As the primary analyses showed no positive effects, the NH-PP-OC population was used, which is characterized by a relatively liberal approach but may introduce selection bias.

Since the analyses were based on health insurance claims data, which are primarily collected for billing purposes, the validity of the data could be limited. Thus, the plausibility and validity of the data had to be checked. Data were not available for all insured NHRs with a long-term care grade from Saarland, which indicates a selection bias. However, data were available from six SHIs that insured the majority of the target population. Corresponding analyses were carried out to check the plausibility and the validity of the data. For example, data from the Federal Statistical Office were used to check whether the ITT population is comparable with the target population of NHRs in Saarland with a long-term care grade, with positive results. Furthermore, the diagnoses required for the calculation of comorbidity were validated internally according to Schubert and colleagues [46]. However, it was not feasible to calculate comorbidity scores for all NHRs, as in a few instances it was not possible to merge diagnosis and NHR characteristics due to technical constraints. Following a review of these cases through descriptive analyses and subgroup analysis, the potential for bias is estimated to be minimal. However, this led to a reduction of the underlying population in models analysing comorbidity as a fixed effect.

Another limiting factor may be the handling of cost data, which could limit the validity of our results. Costs are quarter-based for ambulatory services. Since the start (May 2019) and end (July 2020) of the study did not align with billing quarters, the corresponding costs were considered on a proportionate basis. Implementation costs and the costs of the 25% position in NHs were not considered in the health economic analyses, as they are not reimbursed by SHIs. The costs of the IG could therefore be underestimated.

Overall, some changes have become necessary to the study protocol, e.g., due to delays in recruitment or unexpected occurrences in the data set. The study may be limited by these changes. For transparency, changes to the protocol are listed and explained in S2 Table.

Since the COVID-19 pandemic had no influence on the data collection and both groups were exposed to the pandemic to a comparable extent with similar incidences (see S6 Table), it may be assumed that the pandemic had no significant immediate impact on the results. However, the generalizability of the results may be limited and sophisticated analyses of the impact of the pandemic on the intervention effect deserve separate attention with a distinct research question and corresponding methods.

This study used health insurance claims data to evaluate an interprofessional care concept in NH. The evaluation of complex interventions benefits from the use of different methods and data sources. Both quantitative and qualitative data were collected and analysed to assess the implementation of the new intervention, and thus this study is only able to provide a part of the full picture. Further methods and results deserve separate attention and will be published elsewhere.

## Generalizability

The results can be generalized to other regions in Germany and to comparable care situations in which medical services are provided in NHs to a limited extent. While the largely standardized framework for medical and nursing care in the federal states suggests that the results can be generalized, the sample size may be insufficient to yield significant results. In addition, the target population may be inadequately represented at both the NH level and the NHR level. Although the pragmatic study design enables generalizability, the NHs that participated in the study may not be comparable with the remaining population of NHs in Saarland, as they may be characterized by a higher motivation and willingness to implement the intervention. Part of the implementation of the interprofessional care concept involved relationship building, which takes a considerable amount of time and effort in an understaffed environment with high workload. The generalizability of the results may also be limited as the project was conducted during the COVID-19 pandemic.

## Conclusion

Overall, the interprofessional care concept was not found to have an effect on the overall hospitalization rate of NHRs. A lack of participation in the IG, which was lower than expected at both the NH and NHR level, could be one explanation for our findings. The validity of the results is therefore limited. However, the results of the SaarPHIR project suggest positive tendencies in patient-relevant outcomes, like ACSCs and NHSCs, and was not more expensive for SHIs than routine care, taking into account the available data.

## Supporting information

S1 Fig Flowchart of the claims data provided and in-/exclusion of insured residents in the open analyses populations. Abbreviations: cRCT = Cluster-randomized controlled trial, LOI = Letter of interest, NH-PP-OC = Nursing home per protocol open cohort (subgroup of NH that were actively participating in the intervention group according to the study protocol, while the control group participants remained the same), R-PP-OC = Residents per protocol open cohort (subgroup of NH-PP-OC including intervention group residents only if they received project-specific services).
(TIF)

S1 Table SaarPHIR outcomes overview. Only greyed out rows are subject of the article.
(PDF)

S2 Table Changes of the study design according to protocol. Abbreviations: CG = Control group, IG = Intervention group, NH = Nursing home, NHR = Nursing home residents.
(PDF)

S3 Table Components of the planned intervention in the SaarPHIR project according to TIDieR^1^. ^1^ Hoffmann TC, Glasziou PP, Boutron I*, et al.* Better reporting of interventions: template for intervention description and replication (TIDieR) checklist and guide. BMJ 2014; 348: g1687. Abbreviations: GP = General practitioner, NH = Nursing home.
(PDF)

S4 Table Implementation strategies for the interprofessional care concept according to Proctor et al.1. 1Proctor EK, Powell BJ, McMillen JC. Implementation strategies: recommendations for specifying and reporting. Implementation Science 2013; 8: 139. Abbreviations: IG = Intervention group, NH = Nursing home.
(PDF)

S5 Table Characteristics per study arm and district. Abbreviations: CG = Control group, IG = Intervention group, SD = Standard deviation, NH = Nursing home, NHR = Nursing home residents, hospi. = hospitalizations.
(PDF)

S6 Table Incidence of COVID-19 cases during the SaarPHIR cRCT in the closed ITT population. (PDF)

S7 Table Results of the primary outcome in a district and NH random effect model compared to the primary analysis model. Abbreviations: IRR = Incidence rate ratio, CI = Confidence interval, CG = Control group, IG = Intervention group, SD = Standard deviation, ITT = Intention to treat; The primary endpoint includes all hospitalizations during the observation period (including incident and recurrent hospitalizations). Model 1: Results of the primary analysis, IRR and CI were estimated using Poisson regression in the closed ITT population in the primary observation period. Model 1.i: in contrast to Model 1, IRR and CI were estimated including the district level and the NH level, nested within the districts, as random effects.
(PDF)

S8 Table Results of secondary outcomes in a district and NH random effect model. 1 Please note that the number of residents included in these models differs from the number of residents in the respective populations, as it was not possible to calculate a comorbidity score for all residents. Abbreviations: ACSC = Ambulatory care-sensitive conditions, NHSC = Nursing home-sensitive conditions, IRR = Incidence rate ratio, MRR = Mortality rate ratio, CI = Confidence interval, CG = Control group, IG = Intervention group, cRCT = Cluster-randomized controlled trial, SD = Standard deviation. Model ACSC: includes all (incident and recurrent) hospitalizations due to ACSCs during the cRCT; IRR and CI were estimated in the NH-PP-OC population using Poisson regression including the district level and the NH level, nested within the districts, as random effects. Model NHSC: in contrast to Model ACSCs, hospitalizations due to NHSCs were included; IRR and CI were estimated using negative binomial regression. Model Mortality: the analysis included death from all causes which occurred during the cRCT; MRR and CI were estimated using Poisson regression in the NH-PP-OC population. Model Hospital days: days spent in hospital during the cRCT; effect and CI were estimated using a linear regression in the NH-PP-OC population.
(PDF)
