# Supplementary figures and images for: Effects of an interprofessional care concept in nursing homes evaluated in the SaarPHIR project: A cluster-randomized controlled trial
Source: PLoS One. 2025 May 15;20(5):e0321118. doi: 10.1371/journal.pone.0321118 (PMC12080800; doi:10.1371/journal.pone.0321118)

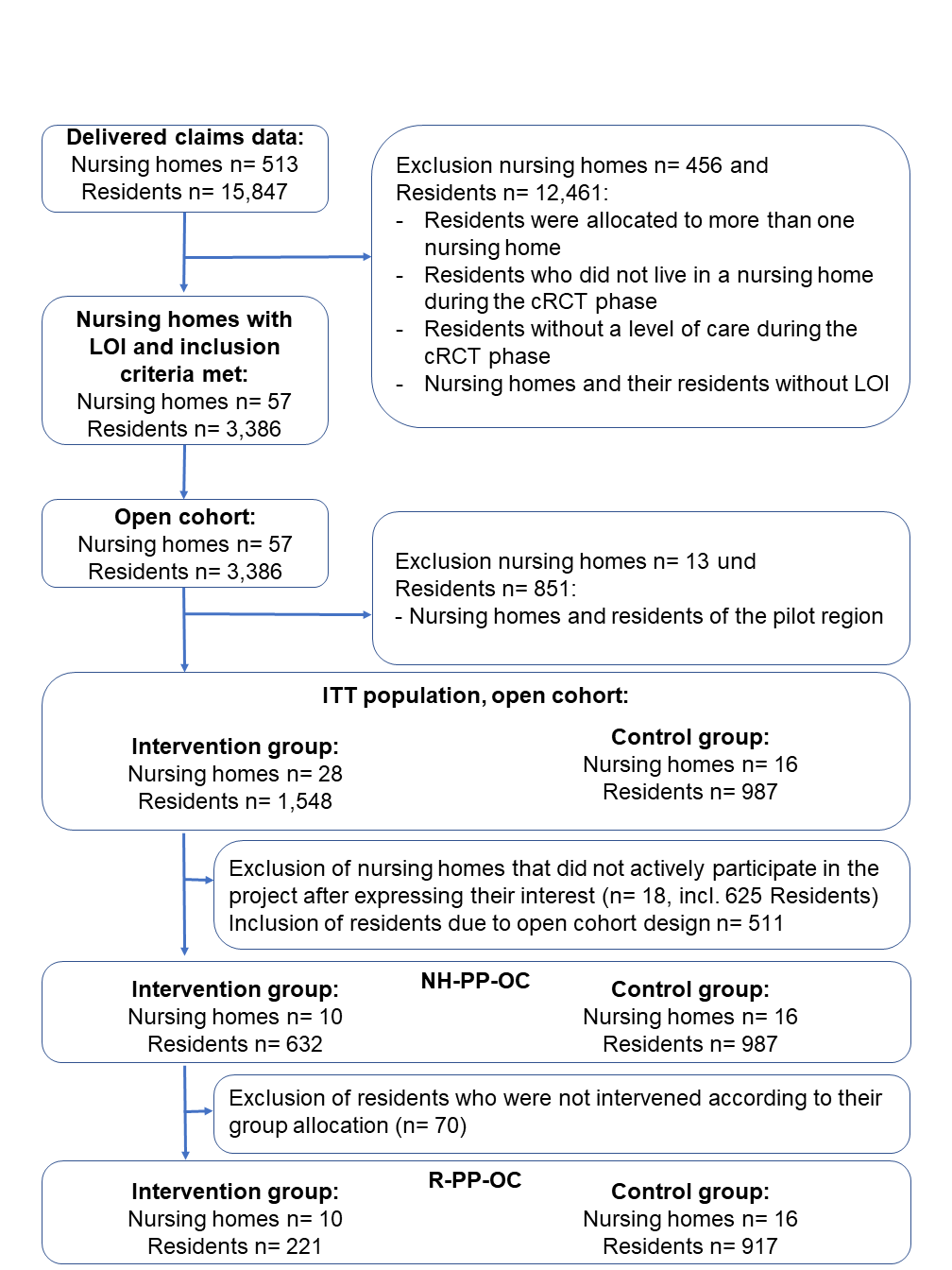

Supplement: S1 Fig — Abbreviations: cRCT = Cluster-randomized controlled trial, LOI = Letter of interest, NH-PP-OC = Nursing home per protocol open cohort (subgroup of NH that were actively participating in the intervention group according to the study protocol, while the control group participants remained the same), R-PP-OC = Residents per protocol open cohort (subgroup of NH-PP-OC including intervention group residents only if they received project-specific services). (TIF) [file pone.0321118.s001.tif]
