# Supplementary material for: Effects of an interprofessional care concept in nursing homes evaluated in the SaarPHIR project: A cluster-randomized controlled trial
Source: PLoS One. 2025 May 15;20(5):e0321118. doi: 10.1371/journal.pone.0321118 (PMC12080800; doi:10.1371/journal.pone.0321118)
Supplement: S1 Table — Only greyed out rows are subject of the article. (PDF) [file pone.0321118.s002.pdf]

S1 Table. SaarPHIR outcomes overview.

Topic	Outcomes	Data Basis
**Effectiveness Analysis**	**Hospitalization**<br>**Ambulatory care sensitive conditions**<br>**Nursing home sensitive conditions**<br>**Hospital days**<br>**Mortality**	**Health claims data**
	Residents'quality of life (Quality of Life in Alzheimer's Disease)	Questionnaire
	Hospital admissions from the residents' point of view	Resident files
**Health Economic Analysis**	**Comparison of total costs between IG (incl. costs provided for intervention services) and CG**	**Health claims data**
**Drug safety**	Medication safety checks: number, scope, and performance	Questionnaire
	Drug supply and use in emergency situations	Questionnaire
**Process Evaluation**	Fidelity	Questionnaire, qualitative interviews
	In-and unintended effects	Questionnaire, qualitative interviews
	Barriers and facilitators	Questionnaire, qualitative interviews
	Context and structures	Questionnaire, qualitative interviews
	Mechanisms of action	Questionnaire, qualitative interviews

Only greyed out rows are subject of the article.
