# Supplementary material for: Effects of an interprofessional care concept in nursing homes evaluated in the SaarPHIR project: A cluster-randomized controlled trial
Source: PLoS One. 2025 May 15;20(5):e0321118. doi: 10.1371/journal.pone.0321118 (PMC12080800; doi:10.1371/journal.pone.0321118)
Supplement: S2 Table — Abbreviations: CG = Control group, IG = Intervention group, NH = Nursing home, NHR = Nursing home residents. (PDF) [file pone.0321118.s003.pdf]

S2 Table. Changes of the study design according to protocol.

Study design according to protocol	Change of the study protocol	Explanation
Start of the cRCT	1-month postponement	Delays during recruitment led to a 1-month postponement from the originally scheduled start (April 2019)
Follow-up of 12 months	Follow-up of 15 months study	The intervention phase was extended by 3 months to 15 months due to pending recruitments and contractual commitments with physicians as well as pandemic-related issues which impeded progress.
Recruitment of nursing homes prior to randomization	Some nursing homes were recruited after randomization	Additional recruitment, as the sample size was not reached at the start of the study.
Inclusion criteria:<br>residents living in NHs, were insured by one of the six participating statutory health insurers (SHIs) and had received a long-term care grade based on their ability to perform activities of daily living.	Inclusion criterion:<br>NHR who lived in a NH at the time during the cRCT phase	The criterion was modified post hoc, given that the data set included NHR who did not live in a NH during the cRCT, but may have done so afterwards.
	Exclusion criterion:<br>NHR who lived in more than one NH during the cRCT	The criterion was defined post hoc, as the data set lacked information regarding the time of entry and exit from a NH. Consequently, the aforementioned NHR could not be assigned to the corresponding NH at the respective time.
Populations for sensitivity analyses:<br>1. Insured residents who live in participating NHs at the start of the study or during the course of the study (open cohort design) (NH-PP-OC)<br>2. An intervention group consisting of only participating insured residents (selective agreement, open cohort design) (R-PP-OC)<br>3. All insured residents in Saarland.	Populations for sensitivity analyses:<br>1.1 Population was realized as planned and named NH-PP-OC<br>1.2 Insured residents who live in participating NHs at the start of the study (closed cohort design) (NH-PP-CC)<br>2.1 Population was realized as planned and named (R-PP-OC)<br>2.2 The population of the IG consisting of only participating insured residents, i.e. who received	In addition to the open cohorts, closed cohorts for the 1st and 2ed population were incorporated to facilitate a comprehensive representation and comparison of the results. The 3rd population was not modelled, given that it can be characterized as relatively conservative and that the primary analysis did not yield any positive effects.

	intervention services (selective agreement). The CG included only NHR in participating NHs who did not receive intervention services. (closed cohort design) (R-PP-CC)<br><br>3. Population was not realized	
Secondary outcomes based on claims data:<br>- Hospitalizations due to ambulatory care-sensitive conditions (ACSC)	Secondary outcomes based on claims data:<br>- Hospitalizations due to nursing home-sensitive conditions (NHSCs)<br>- All-cause death<br>- Hospital days	The outcome NHSCs was included post hoc, due to the specific focus on the medical care of NHR and the overlap between the authors' recommendations and the intervention studied in the SaarPHIR project.<br><br>The outcomes all-cause death and hospital days were included post hoc in order to facilitate comparison of the results with those of similar publications.
Sensitivity analyses with primary and secondary outcomes accounting for clustering at NH level.	Sensitivity analyses with primary and secondary outcomes accounting for clustering at district and NH level.	Under normal circumstances, the randomized cluster level would be adopted for analysis. To avoid a contamination bias, randomization was performed at district level. This means that only six clusters were available for randomization. However, a minimum of eight clusters is recommended for the analysis of a cRCT. Thus, the sample size calculation and the analyses were conducted at the individual level and considered the NH as a cluster level to ensure a pragmatic evaluation of the intervention within the complex healthcare system. This unconventional approach assumed, that the NHs were characterized by the districts in which they operate. To test this assumption, post hoc sensitivity analyses were conducted with primary and

		secondary outcomes that included district as a random effect and NHs as a nested random effect within districts.
Health economic analyses include a cost-effectiveness analysis and incremental cost-effectiveness ratios (ICER)	Costs were analysed descriptively and exploratively	A cost-effectiveness analysis and incremental cost-effectiveness ratios (ICER) were not applied because the intervention did not show a significant positive. However, in order to report the health economic aspects in a transparent way, the costs were analysed descriptively and exploratively.
Population of sensitivity analyses in health economic evaluation	Sensitivity analyses were not realized	Sensitivity analyses were planned as part of the ICER calculation. The ICER calculation was not realized, given that the primary analysis did not yield any positive effects. Thus, the planned sensitivity analyses were not performed.
