# Supplementary material for: Effects of an interprofessional care concept in nursing homes evaluated in the SaarPHIR project: A cluster-randomized controlled trial
Source: PLoS One. 2025 May 15;20(5):e0321118. doi: 10.1371/journal.pone.0321118 (PMC12080800; doi:10.1371/journal.pone.0321118)
Supplement: S3 Table — 1 Hoffmann TC, Glasziou PP, Boutron I, et al. Better reporting of interventions: template for intervention description and replication (TIDieR) checklist and guide. BMJ 2014; 348: g1687. Abbreviations: GP = General practitioner, NH = Nursing home. (PDF) [file pone.0321118.s004.pdf]

S3 Table. Components of the planned intervention in the SaarPHIR project according to TIDieR[1].

Brief name	Why?	What?	Who provided?	How?	Where?	When and how much?
**Reorganization of physicians**	The reorganization of physicians is a structural requirement for the planning of a joint care routine, e.g. on-call duties and pre-weekend visits.	The reorganization of GPs refers to the formation of medical care teams that jointly plan a care routine (e.g. on-call duties and pre-weekend visits) for a NH and its residents. Furthermore, each medical care team selects a representative of the team and develop a consultation schedule for a particular NH. .	Care by a reorganized team of GPs is provided by participating GPs who practise in the area of a participating NH.	Reorganization is realized by enabling communication between GPs virtually, face to face or by telephone. GPs are responsible for their communication and organization within the medical care team. Consultations in NHs follow a pre-planned consultation schedule. Patients are treated by the doctor on duty, without any claim to a specific treatment.	In the catchment area of a NH.	The teams primarily consist of three to five physicians, who visit their collaborating NHs at least once a week.

**Extended on-call duty**	The extended on-call duty ensures increased availability of GPs for nurses in order to avoid critical situations which could lead to hospitalization at the weekend.	The medical care teams provide extended on-call duty hours for the cooperating NH to address or check e.g. changes in the residents' state of health which occur out of regular consultation hours.	Each GP of each medical care team performs an on-call duty in accordance with the duty roster their team provided.	A duty roster for each quarter is drawn up 4 weeks in advance and provided to the NH staff by the medical care teams.	Services are provided in every NH collaborating with a medical care team.	A GP from the care team is on duty from Monday to Friday, from 6 pm to 9 pm.
**Pre-weekend visits**	The purpose of pre-weekend visits is to reconcile detected changes in health status of NHRs and avoid critical situations which could lead to hospitalization at the weekend.	A regular pre-weekend visit in the form of a Friday afternoon or Saturday visit is ensured.	The pre-weekend visits are provided by the medical care team.	These visits follow certain organizational rules. First, NH staff list who is to be visited and compile all relevant information. Only acute cases are to be covered. GPs are informed about the cases prior to their visit. The pre-weekend visit is not intended to replace any physician’s regular visit during the week.	Services are provided in every NH collaborating with a medical care team.	Every Friday afternoon or Saturday if necessary.

**Screenings**	The screenings are implemented to detect changes in health status of NHRs at an early stage and avoid critical situations such as hospitalization.	Part of the intervention is the regular performance of screenings and assessments. Core assessments include basic geriatric screening (Lachs screening) and fall prevention. Further assessments evaluate nutrition/hydration, dementia, depression/anxiety, and activities of daily living or mobility).	Screenings are conducted by nurses employed in participating NHs.	The nurses conduct the screenings and assessments with the residents in their rooms (face to face). The nurses are supported by forms and descriptions in the reference manual. Each screening must be documented in the resident file, and the completed form must be attached.	Services are provided in every NH collaborating with a medical care team.	Core screenings are mandatory on admission to the NH, and once a year thereafter. Further assessments can be carried out if relevant.
