# Supplementary material for: Effects of an interprofessional care concept in nursing homes evaluated in the SaarPHIR project: A cluster-randomized controlled trial
Source: PLoS One. 2025 May 15;20(5):e0321118. doi: 10.1371/journal.pone.0321118 (PMC12080800; doi:10.1371/journal.pone.0321118)
Supplement: S4 Table — 1Proctor EK, Powell BJ, McMillen JC. Implementation strategies: recommendations for specifying and reporting. Implementation Science 2013; 8: 139. Abbreviations: IG = Intervention group, NH = Nursing home. (PDF) [file pone.0321118.s005.pdf]

S4 Table. Implementation strategies for the interprofessional care concept according to Proctor et al.1.

	INFORMATION EVENTS	ON-SITE MEETINGS	TELEPHONE SUPPORT	GUIDEBOOK	TEAM MEETINGS	PROJECT FUNDS FOR COORDINATING NURSE	BILLABLE SERVICE FEES
**Actor(s)**	The Saarland Association of Statutory Health Insurance Physicians and the Association of Care	The Saarland Association of Statutory Health Insurance Physicians and the Association of Care	The Saarland Association of Statutory Health Insurance Physicians and the Association of Care	The Saarland Association of Statutory Health Insurance Physicians and the Association of Care	Team meetings are provided by the designated coordinating nurse (plus deputy).	The coordinating nurse is recruited in the participating NH of the IG. The funds are transferred to the NH by the consortium lead of the project.	The Saarland Association of Statutory Health Insurance Physicians
**Actions(s)**	Information events are offered regionally and in person, explaining all details regarding the intervention and its implementation.	On-site meetings are offered, explaining all details regarding the intervention and its implementation.	Telephone support is offered, including clarification of queries and problems regarding the intervention and its implementation.	A guidebook summarizing the components of the SaarPHIR intervention, providing information and templates of the relevant screening and assessment instruments.	Team meetings provide the forum for an interprofessional exchange between the medical care team and the responsible nursing staff on organisational and medical topics.	The NH designates one nurse who is entrusted with a coordinating function. A part-time position for each facility participating in the intervention is funded.	Physicians bill for their provided SaarPHIR services.
**Target(s) of action**	Participating physicians and NHs of the IG	Participating NHs (if desired) of the IG	Participating physicians and NHs (if desired) of the IG	Participating NHs of the IG	Participating physicians and coordinating nurse of the IG	Participating NHs of the IG	Participating physicians of the IG
**Temporality**	At the beginning of intervention phase	At the beginning of the intervention phase and throughout the implementation process	At the beginning of the intervention phase and throughout the implementation process	At the beginning and throughout the intervention phase	At the beginning and throughout the intervention phase	At the beginning and throughout the intervention phase	At the beginning and throughout the intervention phase

**Dose**	One-time initial event per region, supplemented if needed	Support is arranged as required	Support is arranged as required	Initial setup of material, supplemented if needed	Team meetings are held regularly (at least four times a year).	The project funds one part-time position (25%) for each NH.	The project funds all provided services
**Implementation outcome(s) affected**	Adoption, implementation (fidelity)	Adoption, implementation (fidelity) sustainability	Adoption, implementation (fidelity) sustainability	Adoption, implementation (fidelity), sustainability	Implementation (fidelity)	Adoption, implementation (costs)	Adoption, implementation (costs)
**Justification**	The information events are employed to promote understanding and implementation of the intervention components.	The on-site meetings are employed to promote understanding and implementation of the intervention components.	The telephone support is employed to support the implementation of the intervention components in case of questions or difficulties.	Educational materials are important to support the NH staff learning how to deliver the intervention, which may influence the uptake, delivery and maintenance of the intervention. Distributing educational materials promotes self-efficacy and a positive attitude towards the intervention and supports the establishment of a pro-implementation attitude.	Team meetings are introduced to promote interprofessional cooperation and structured exchange, e.g. exchanging medical information, discussing cases, and jointly solving problems in crisis situations.	Implementing a new model of care is a highly complex process of change in the routine care of the NHs, which are subject to financial pressure. The coordinating nurse is necessary to manage the interprofessional exchange and financial support is necessary to support the adoption, to optimize the implementation and prepare its maintenance of new interventions like SaarPHIR.	Implementing a new model of care is a highly complex process of change in the routine care of the physicians, who are subject to financial pressure. Financial support is necessary to support the adoption of new interventions like SaarPHIR, to optimize the implementation and prepare its maintenance.
