# Supplementary material for: Effects of an interprofessional care concept in nursing homes evaluated in the SaarPHIR project: A cluster-randomized controlled trial
Source: PLoS One. 2025 May 15;20(5):e0321118. doi: 10.1371/journal.pone.0321118 (PMC12080800; doi:10.1371/journal.pone.0321118)
Supplement: S5 Table — Abbreviations: CG = Control group, IG = Intervention group, SD = Standard deviation, NH = Nursing home, NHR = Nursing home residents, hospi. = hospitalizations. (PDF) [file pone.0321118.s006.pdf]

S5 Table. Characteristics per study arm and district.

Study arm	Districts	NH [N]	NHR [N]	Age [Mean [SD]]	Average no. of hospi. before cRCT [Mean [SD]]	Person-years observed in cRCT [Mean [SD]]
CG	Landkreis Merzig-Wadern	7	304	80 [11]	1.02 [1.38]	1.08 [0.33]
	Landkreis St. Wendel	3	80	82 [10]	1.31 [1.56]	0.97 [0.41]
	Regionalverband Saarbruecken	6	296	80 [11]	0.99 [1.46]	1.01 [0.38]
IG	Landkreis Neunkirchen	6	152	81 [11]	1.04 [1.59]	1.06 [0.33]
	Landkreis Saarlouis	12	461	84 [10]	1.15 [1.40]	1.02 [0.37]
	Saar-Pfalz-Kreis	10	440	82 [10]	0.83 [1.19]	1.01 [0.38]
