# Supplementary material for: Effects of an interprofessional care concept in nursing homes evaluated in the SaarPHIR project: A cluster-randomized controlled trial
Source: PLoS One. 2025 May 15;20(5):e0321118. doi: 10.1371/journal.pone.0321118 (PMC12080800; doi:10.1371/journal.pone.0321118)
Supplement: S6 Table — (PDF) [file pone.0321118.s007.pdf]

S6 Table. Incidence of COVID-19 cases during the SaarPHIR cRCT in the closed ITT population.

	**Overall**<br>N = 1,733	**IG**<br>N = 1,053	**CG**<br>N = 680
**COVID-19 cases**	129 (7.4)	76 (7.2)	53 (7.8)

COVID-19 was identified, if the ICD-10 Codes U07.1 or U07.2 was coded in in- or outpatient care claims data between May 2019 and July 2020.
