# Supplementary material for: Effects of an interprofessional care concept in nursing homes evaluated in the SaarPHIR project: A cluster-randomized controlled trial
Source: PLoS One. 2025 May 15;20(5):e0321118. doi: 10.1371/journal.pone.0321118 (PMC12080800; doi:10.1371/journal.pone.0321118)
Supplement: S7 Table — Abbreviations: IRR = Incidence rate ratio, CI = Confidence interval, CG = Control group, IG = Intervention group, SD = Standard deviation, ITT = Intention to treat; The primary endpoint includes all hospitalizations during the observation period (including incident and recurrent hospitalizations). Model 1: Results of the primary analysis, IRR and CI were estimated using Poisson regression in the closed ITT population in the primary observation period. Model 1.i: in contrast to Model 1, IRR and CI were estimated including the district level and the NH level, nested within the districts, as random effects. (PDF) [file pone.0321118.s008.pdf]

S7 Table. Results of the primary outcome in a district and NH random effect model compared to the primary analysis model.

	Model 1			Model 1.i		
**Hospitalization**	**IRR**	**95% CI**	**p-value**	**IRR**	**95% CI**	**p-value**
**(Intercept)**	0.00	0.00, 0.00	<0.001	0.00	0.00, 0.00	<0.001
**CG**	—	—		—	—	
**IG**	0.94	0.78, 1.14	0.5	0.94	0.76, 1.16	0.5
**Hospitalization before cRCT**	1.31	1.27, 1.34	<0.001	1.31	1.27, 1.34	<0.001
**Random effect NH**						
Intercept SD	0.25				0.24	
**Random effect district**						
Intercept SD					0.06	
