# Supplementary material for: Effects of an interprofessional care concept in nursing homes evaluated in the SaarPHIR project: A cluster-randomized controlled trial
Source: PLoS One. 2025 May 15;20(5):e0321118. doi: 10.1371/journal.pone.0321118 (PMC12080800; doi:10.1371/journal.pone.0321118)
Supplement: S8 Table — 1 Please note that the number of residents included in these models differs from the number of residents in the respective populations, as it was not possible to calculate a comorbidity score for all residents. Abbreviations: ACSC = Ambulatory care-sensitive conditions, NHSC = Nursing home-sensitive conditions, IRR = Incidence rate ratio, MRR = Mortality rate ratio, CI = Confidence interval, CG = Control group, IG = Intervention group, cRCT = Cluster-randomized controlled trial, SD = Standard deviation. Model ACSC: includes all (incident and recurrent) hospitalizations due to ACSCs during the cRCT; IRR and CI were estimated in the NH-PP-OC population using Poisson regression including the district level and the NH level, nested within the districts, as random effects. Model NHSC: in contrast to Model ACSCs, hospitalizations due to NHSCs were included; IRR and CI were estimated using negative binomial regression. Model Mortality: the analysis included death from all causes which occurred during the cRCT; MRR and CI were estimated using Poisson regression in the NH-PP-OC population. Model Hospital days: days spent in hospital during the cRCT; effect and CI were estimated using a linear regression in the NH-PP-OC population. (PDF) [file pone.0321118.s009.pdf]

S8 Table. Results of secondary outcomes in a district and NH random effect model.

Outcomes	ACSC<br>NH: N = 26<br>NHR: N = 1,566[1]			NHSC<br>NH: N = 26<br>NHR: N = 1,566[1]			Mortality<br>NH: N = 26<br>NHR: N = 1,566[1]			Hospital days<br>NH: N = 26<br>NHR: N = 1,566[1]		
	**IRR**	**95% CI**	**p-value**	**IRR**	**95% CI**	**p-value**	**MRR**	**95% CI**	**p-value**	**Effect**	**95% CI**	**p-value**
**(Intercept)**	0.00	0.00, 0.00	<0.001	0.00	0.00, 0.00	<0.001	0.00	0.00, 0.00	<0.001	5.3	3.2, 7.3	<0.001
**CG**	—	—		—	—		—	—		—	—	
**IG**	0.85	0.64, 1.14	0.3	0.75	0.57, 0.98	0.037	1.01	0.70, 1.46	>0.9	-0.24	-2.9, 2.4	0.8
**Hospitalization before cRCT**	1.23	1.19, 1.27	<0.001	1.33	1.26, 1.42	<0.001	1.12	1.06, 1.17	<0.001	1.7	1.2, 2.1	<0.001
**Sex**												
**Male**	—	—		—	—		—	—		—	—	
**Female**	0.73	0.61, 0.88	<0.001	0.73	0.58, 0.91	0.005	0.63	0.52, 0.77	<0.001	-1.1	-2.6, 0.40	0.15
**Age**	1.01	1.00, 1.02	0.014	1.02	1.01, 1.03	0.003	1.06	1.04, 1.07	<0.001	-0.07	-0.14, 0.00	0.042
**Charlson comorbidity index**	1.09	1.06, 1.12	<0.001	1.07	1.03, 1.11	<0.001	1.04	1.00, 1.07	0.043	0.20	-0.05, 0.44	0.12
**Random effect NH** Intercept *SD*	0.19			0.11			0.21			0.98		
**Random effect district** Intercept *SD*	0.10			0.08			0.15			0.65		
**Residual *SD***										14		
